# ERK1/ATF-2 signaling axis contributes to interleukin-1β-induced MMP-3 expression in dermal fibroblasts

**DOI:** 10.1371/journal.pone.0222869

**Published:** 2019-09-19

**Authors:** Nanako Kitanaka, Rei Nakano, Manabu Sakai, Taku Kitanaka, Shinichi Namba, Tadayoshi Konno, Tomohiro Nakayama, Hiroshi Sugiya

**Affiliations:** 1 Laboratories of Veterinary Biochemistry, 3 Veterinary Internal Medicine, and 4Veterinary Radiotherapy, Nihon University College of Bioresource Sciences, Kameino, Fujisawa, Kanagawa, Japan; 2 Laboratory for Cellular Function Conversion Technology, RIKEN Center for Integrative Medical Sciences, Suehiro-cho, Tsurumi, Yokohama, Kanagawa, Japan; 3 Laboratories of Veterinary Internal Medicine, Nihon University College of Bioresource Sciences, Kameino, Fujisawa, Kanagawa, Japan; 4 Laboratories of Veterinary Radiotherapy, Nihon University College of Bioresource Sciences, Kameino, Fujisawa, Kanagawa, Japan; China Medical University, TAIWAN

## Abstract

Matrix metalloproteinases (MMPs) play a pivotal role in tissue remodeling by degrading the extracellular matrix (ECM) components. This mechanism is implicated in a variety of physiological and pathological cellular processes including wound healing. One of the key proteins involved in this process is the proinflammatory cytokine interleukin-1β (IL-1β, which induces the expression of MMP-3 mRNA and the secretion of MMP-3 protein by dermal fibroblasts. In this study, we first investigated the contribution of activating transcription factor 2 (ATF-2) to IL-1β-induced MMP-3 expression in dermal fibroblasts. Our results showed that in cells transfected with ATF-2 siRNA or treated with the ATF-2 inhibitor SBI-0087702, IL-1β-induced MMP-3 mRNA expression was reduced. We also demonstrated that IL-1β stimulates the phosphorylation of ATF-2. These observations suggest that ATF-2 plays an important role in IL-1β-induced MMP-3 expression. Next, we investigated the role of MAPK signaling in ATF-2 activation. In cells treated with the extracellular signal-regulated kinase (ERK) inhibitor FR180240, as well as in cells transfected with ERK1 and ERK2 siRNAs, IL-1β-induced MMP-3 mRNA expression was reduced. In addition, we showed that IL-1β induced the phosphorylation of ERK1/2. These observations suggest that ERK1 and ERK2 are involved in IL-1β-induced MMP-3 expression. However, ERK1 and ERK2 do seem to play different roles. While the ERK inhibitor FR180204 inhibited IL-1β-induced ATF-2 phosphorylation, only in cells transfected with ERK1 siRNA, but not ERK2 siRNA, IL-1β-induced ATF-2 phosphorylation was reduced. These findings suggest that the ERK1/ATF-2 signaling axis contributes to IL-1β-induced MMP-3 expression in dermal fibroblasts.

## Introduction

Wound healing is a complex biological process consisting of inflammation, the formation of granulation tissues, re-epithelialization, matrix formation, and remodeling [1, 2]. Numerous cytokines and growth factors, which contribute to the regulation of wound healing [2], are synthesized and secreted in several kinds of cells such as inflammatory cells, keratinocytes, and fibroblasts in the wounded skin [2]. Mesenchymal-epithelial interactions are considered important for epithelial homeostasis and regeneration. In the skin, the communication between keratinocytes and cells of mesenchyme, predominantly dermal fibroblasts, is involved in wound healing [3–5]. For example, keratinocyte-derived cytokines have been demonstrated to provoke the expression of genes involved in the process of wound healing in fibroblasts [3, 5, 6].

Interleukin (IL)-1β is a regulatory factor implicated in wound healing. After skin injury, polymorphonuclear leukocytes, macrophages, and keratinocytes at the wound edges release IL-1β, which functions in concert with other soluble factors such as transforming growth factor (TGF)-β and various chemokines to induce wound repair [1, 2, 7, 8]. Recently, the inflammasome, a multiprotein complex of the innate immune system responsible for the activation and release of IL-1β from several skin cell types, has been implicated in the development of wounds [9].

Matrix metalloproteinases (MMPs), a family of zinc-dependent and neutral endopeptidases, play an important role in the process of tissue remodeling by degrading the extracellular matrix (ECM) components [10, 11]. In addition, MMPs have been implicated in a variety of normal and pathological cellular processes [10, 11] including wound healing [12, 13]. MMPs can be classified broadly by substrate specificity into collagenases, gelatinases, stromelysins, elastases, membrane-type, and other MMPs [10, 14, 15]. Of these, MMP-3 (stromelysin-1) degrades a broad array of extracellular substrates such as proteoglycan, laminin, fibronectin, and the non-fibrillar collagens [16]. MMP-3 has been reported to be involved in skin wound healing [17–22]. Basal expression and activities of MMPs are usually low in normal physiological condition. It has been considered that, in response to various stimuli (e.g., inflammatory cytokines and growth factors), the expression of MMPs is induced, which is primarily regulated at the transcriptional level via tissue/cell-specific intracellular signaling pathways [23–25].

Activating transcription factor 2 (ATF-2) is a member of the ATF/cyclic AMP-responsive element binding protein (CREB) family of transcription factors, which contains the basic/leucine zipper motifs (bZIP), and is involved in inflammation by regulating gene expression [26, 27]. ATF-2 has been demonstrated to contribute to the regulation of expression of MMPs such as MMP-1 [28, 29], MMP-2 [30], MMP-3 [31], MMP-9 [32] and MMP-13 [33]. In the previous studies, it has been demonstrated that IL-1β induces MMP-3 expression in dermal fibroblasts [34–36]. However, the contribution and regulation of ATF-2 in the MMP-3 expression is not fully characterized.

In this study, we demonstrate the contribution of ATF-2 activation via the extracellular-regulated kinase 1 (ERK1) signaling to IL-1β-induced MMP-3 expression in dermal fibroblasts.

## Materials and methods

### Materials

Canine recombinant IL-1β was purchased from Kingfisher Biotech, Inc. (Saint Paul, MN). TRIzol, Lipofectamine 2000 and Opti-MEM were purchased from Life Technologies Co. (Carlsbad, CA). PrimeScript RT Master Mix, SYBR Premix Ex Taq II, Thermal Cycler Dice Real Time System II, TP900 Dice Real Time v4.02B, and CELLBANKER 1 plus medium were obtained from TaKaRa Bio Inc. (Shiga, Japan). Rabbit monoclonal antibodies against phospho-ERK1/2 (p-ERK1/2, D13.14.4E), total-ERK1/2 (t-ERK1/2, 137F5), phospho-ATF-2 (p-ATF-2), and total ATF-2 (t-ATF-2, 20F1) were purchased from Cell Signaling Technology Japan, K.K. (Tokyo, Japan). Horseradish peroxidase-conjugated (HRP-conjugated), anti-rabbit IgG antibodies, ECL Western Blotting Analysis System, and ImageQuant LAS 4000 mini, were purchased from GE Healthcare (Piscataway, NJ). Mini-PROTEAN TGX gel and polyvinylidene difluoride (PVDF) membranes were obtained from Bio-Rad (Hercules, CA). Complete mini EDTA-free protease inhibitor mixture and Block Ace were purchased from Roche (Mannheim, Germany). α-Modified Eagle Minimum essential medium (α-MEM), phenylmethanesulfonyl fluoride (PMSF), sodium fluoride, 4-(2-hydroxyethyl)-1-piperazineethanesulfonic acid (HEPES), and 4-aminophenylmercuric acetate were purchased from Wako Pure Chemical Industries, Ltd. (Osaka, Japan). Fetal bovine serum (FBS) was obtained from Biowest (France). SB239063, FR180204, SP600125 and SBI-0087702 were purchased from Sigma-Aldrich Inc. (St Louis, MO). SensoLyte 520 MMP-3 Assay Kit was purchased from AnaSpec EGT Co. (Fremont, CA). StatMate IV was purchased from ATMS (Tokyo, Japan).

### Cell culture

This study was approved by Nihon University Animal Care and Use Committee (AP13B051). All the experiments were performed in accordance with the relevant guidelines and regulations. Dog dorsal skin samples (n = 3, healthy 3-year-old beagle dogs, male) were collected after local anesthesia with 1% lidocaine and 10 μg/mL adrenaline. To minimize potential pain and infection, remifentanil hydrochloride (3 to 5 μg/kg/min; Janssen Pharmaceutical K.K, Tokyo, Japan) and cefazolin (22 mg/kg; Nichi-Iko Pharmaceutical Co., Ltd, Toyama, Japan) were administered intravenously before the time of awakening after anesthesia. Dermal fibroblasts were isolated by explant culture as reported previously [37]. Briefly, canine dermis from the dorsal skin was collected and cut into 3-mm^2^ sections. Each explant was placed into 90-mm Petri dish. The attached explants were maintained in α-MEM supplemented with 10% FBS, inside a static-culture incubator at 5% CO_2_ and 37°C. The medium was changed once a week and dermal fibroblasts were obtained as outgrowth cells. The cells were harvested using 0.25% trypsin-EDTA once they reached 90–95% confluence. The collected cells were suspended using CELLBANKER 1 supplemented with medium at a density of 2×10^6^ cells/500 μL, and 500 μL of the cell suspension was placed into a sterilized serum tube. The tubes were then placed into a freezing vessel and cryopreserved at -80°C. Before experiments, serum tubes were removed from the freezing vessel and immersed into a water bath at 37°C. The thawed-out cell suspension was transferred into a centrifuge tube containing α-MEM supplemented with 10% FBS, and then centrifuged at 300 *g* for 3 min. After removal of the supernatant, the pellet was suspended in α-MEM 10% FBS and transferred into a 75-cm^2^ culture flask. Static cultures were then maintained under the same conditions as before the cryopreservation. Cells were harvested using 0.25% trypsin-EDTA once they reached approximately 90% confluency. Then, the detached cells were seeded at a density of 1×10^6^ cells/75-cm^2^ culture flask. The fourth-passaged canine dermal fibroblasts were used for all following experiments. Cells from different animals were used in different experiments. Cells were characterized by detecting the mRNA expression of chemotropic factors such as: Netrin-1, Netrin-3, Ephrin-A3, Ephrin-A4, and Semaphorin-4D as reported previously [37]. The mRNA expression of chemotropic factors in dermal fibroblasts was lower compared to mesenchymal stem cells, confirming that the cells are dermal fibroblasts.

### Real-time RT-PCR

Total RNA was extracted from canine dermal fibroblasts with TRIzol reagent. The first-strand cDNA synthesis was carried out with 500 ng of total RNA using PrimeScript RT Master Mix. Real-time RT-PCR was performed with 2 μL of the first-strand cDNA in 25 μL (total reaction volume) with SYBR Premix Ex Taq II. Primers were designed for canine MMP-3, while the house keeping protein TATA box binding protein (TBP), was used as a control. Table 1 shows the sequences of primers used for real-time RT-PCR. Real-time RT-PCR of no-template controls was performed with 2 μL of RNase- and DNA-free water. In addition, real-time PCR of no-reverse transcription control was performed with 2 μL of each RNA sample. All PCR reactions were conducted using Thermal Cycler Dice Real Time System II with the following protocol: 1 cycle of denaturation at 95°C for 30 s, 40 cycles of denaturation at 95°C for 5 s and annealing/extension at 60°C for 30 s. The results were analyzed by the second derivative maximum method and the comparative cycle threshold (ΔΔCt) method using the real-time RT-PCR analysis software. The amplification of TBP from the same amount of cDNA was used as an endogenous control, while cDNA amplification from canine dermal fibroblasts at time 0 was used as a calibration standard.

**Table 1:** Primers used for Real-time RT-PCR.

Gene Name	Gene bank ID	Primer sequences
*MMP-3*	NM_001002967.1	F: 5ʹ- TGACGATGATGAACAATGGACAAG-3ʹ
		R: 5ʹ- GCTAGGGTCAGCCGAGTGAAAG-3ʹ
*TBP*	XM_863452	F: 5'-ACTGTTGGTGGGTCAGCACAAG-3'
		R: 5'-ATGGTGTGTACGGGAGCCAAG-3'

### Western blotting

The cells were lysed with a lysis buffer containing 20 mM HEPES, 1 mM PMSF, 10 mM sodium fluoride, and a complete mini EDTA-free protease inhibitor cocktail at pH 7.4. Protein concentrations were adjusted using the Bradford method [38]. Extracted proteins were boiled at 95°C for 5 min in SDS buffer. Samples were loaded into separate lanes of 12% Mini-PROTEAN TGX gel and separated by electrophoresis. Separated proteins were transferred to PVDF membranes, treated with Block Ace for 50 min at room temperature, and incubated with primary antibodies [p-ERK1/2 (1:1000), t-ERK1/2 (1:1000), p-ATF-2 (1:1000), t-ATF-2 (1:1000)] for 120 min at room temperature. After washing, the membranes were incubated with an HRP-conjugated anti-rabbit or a mouse IgG antibody (1:10000) for 90 min at room temperature. Immunoreactivity was detected using ECL Western Blotting Analysis System. Chemiluminescent signals of the membranes were measured using ImageQuant LAS 4000 mini.

### MMP-3 activity assay

Canine dermal fibroblasts were seeded at a density of 3.0×10^5^ cells per well in 6-well culture plates. The cells were treated with IL-1β after starvation for 24 h, and culture supernatants were collected. To activate pro-MMP, samples were incubated with 4-aminophenylmercuric acetate at 37°C for 24 h. MMP-3 activity in the culture supernatant were measured using the MMP-3 activity kit according to the manufacturer’s instructions. The activity of MMP-3 was detected by a fluorescence microplate reader (Fluoroskan Ascent FL, Thermo Fisher Scientific K.K., Kanagawa, Japan) at excitation/emission wavelengths of 360 nm/460 nm.

### siRNA transfection

Dermal fibroblasts seeded at a density of 1×10^5^ cells/35-mm dish or 5 × 10^5^ cells/90-mm dish, were transfected using Opti-MEM containing 5 μl/mL Lipofectamine 2000 and 50 nM ATF-2 or scrambled siRNA for 6 h [37]. The sequences of the siRNA are indicated in the Supplementary Table 2. The efficiency of siRNA was confirmed by western blotting.

**Table 2:** Sequences for siRNA transfection.

Gene Name	Gene bank ID	siRNA sequences
*ATF-2*	XM_005640334.2	F: 5'-GUCCAUUUGAGAAUGAAUU-3'
		R: 5'-AAUUCAUUCUCAAAUGGAC-3'
*ERK1*	NM_001252035.1	F: 5'-CCAAUGUGCUCCACCGGGA-3'
		R: 5'-UCCCGGUGGAGCACAUUGG-3'
*ERK2*	NM_001110800.1	F: 5'-CCCAAAUGCUGACUCGAAA-3'
		R: 5'-UUUCGAGUCAGCAUUUGGG-3'

### Wound healing assay

Culture-Insert 2 Well (ibidi GmbH, Am Klopferspitz, Germany) was placed on the 35-mm dish. 70 μl of cell suspension (1×10^6^/ml) were applied into each well of the Culture-Insert 2 Well. After the incubation for 24 h, cell culture inserts were carefully removed followed by stimulation of IL-1β. The phase-contrast images were analyzed using the MRI Wound Healing Tool for ImageJ [39].

### Statistical analysis

The data from these experiments are presented as the mean ± standard error of measurement. Statistical analysis was performed using StatMate IV. The data from the time course study were analyzed using two-way analysis of variance, and the data from other experiments were analyzed using one-way analysis of variance. Tukey’s test was used as post-hoc analysis. *P*-values less than 0.05 were considered statistically significant.

## Results

### IL-1β induces MMP-3 expression and secretion

**Fig 1:**
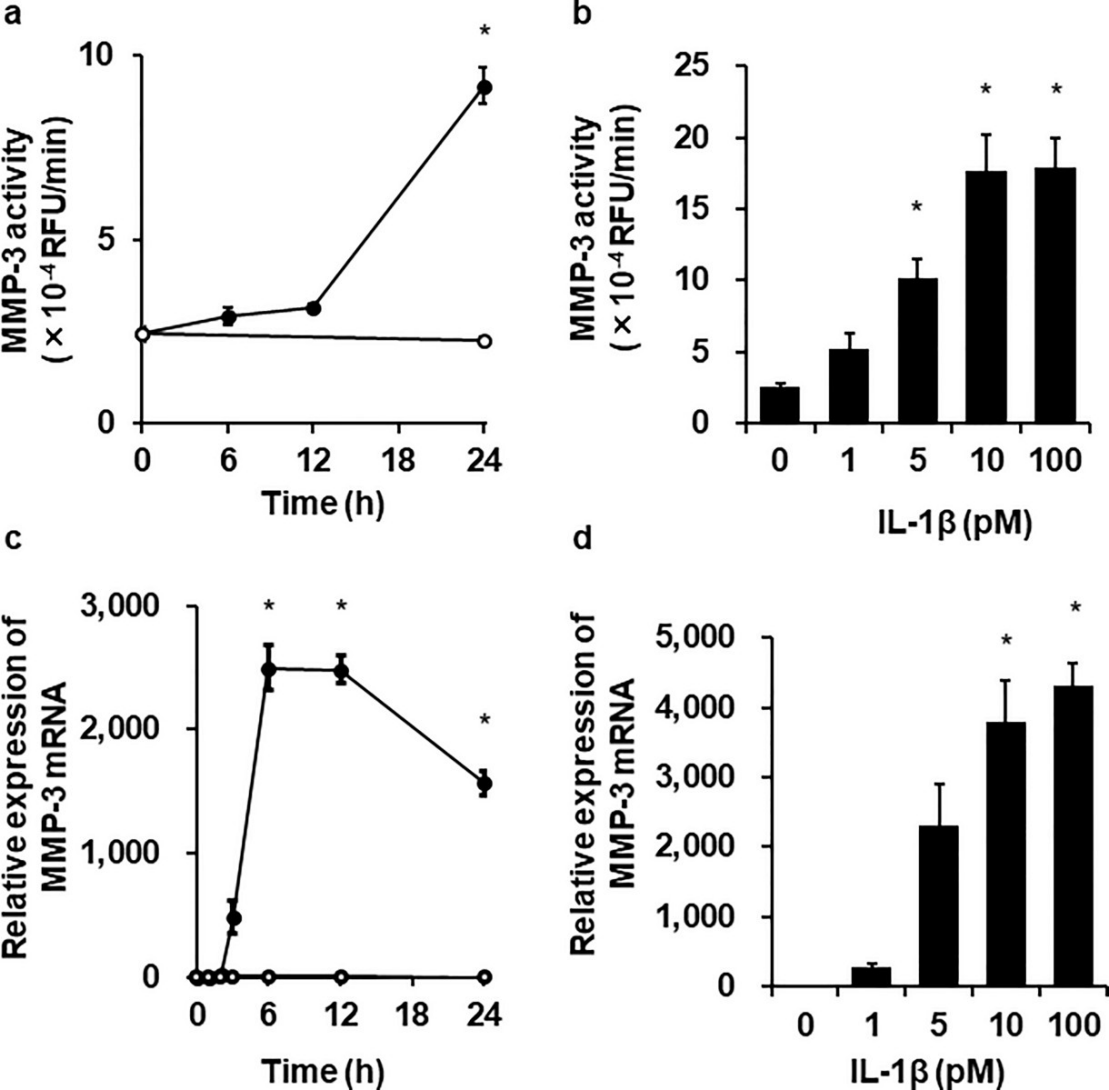
Time- and dose-dependent IL-1β-induced MMP-3 protein secretion and mRNA expression in dermal fibroblasts. The cells were incubated with (closed circle) or without (open circle) 100 pM IL-1β for indicated time (**a, c**), or with indicated concentrations of IL-1β for 24 h (**b**) or 6 h (**d**). At the end of the incubation, protein secretion and mRNA expression of MMP-3 were detected by ELISA and real-time RT-PCR, respectively. TBP was used as an internal standard. Values are expressed as the mean ± SE of 3 independent experiments. The F values were 176.78 (**a**), 20.55 (**b**), 129.23 (**c**) and 22.73 (**d**). The degrees of freedom were 3 (**a**), 4 (**b**), 6 (**c**) and 4 (**d**). *P < 0.05, compared with 0 h (**a, c**), 0 pM (**b, d**).

We first examined the effect of the inflammatory cytokine IL-1β on MMP-3 protein secretion in dermal fibroblasts. Cells treated with IL-1β (100 pM) for 24 h showed a time-dependent increase in MMP-3 activity in the culture medium (Fig 1A). The cells treated with various concentration of IL-1β (0–100 pM) for 24 h showed a dose-dependent increase in MMP-3 activity in the culture medium (Fig 1B). Then, we examined the mRNA expression of MMP-3 using real-time RT-PCR. Incubation with 100 pM IL-1β enhanced MMP-3 mRNA expression in a time-dependent manner (Fig 1C) with the maximum effect observed at 6 h, followed by a decrease in the MMP-3 mRNA expression. The cells treated with various concentration of IL-1β (0–100 pM) for 6 h showed a dose-dependent induction of the MMP-3 mRNA expression (Fig 1D), which was similar to the effect of IL-1β on MMP-3 secretion. These observations suggest that IL-1β stimulated MMP-3 secretion by upregulating MMP-3 expression in canine dermal fibroblasts.

**Fig 2:**
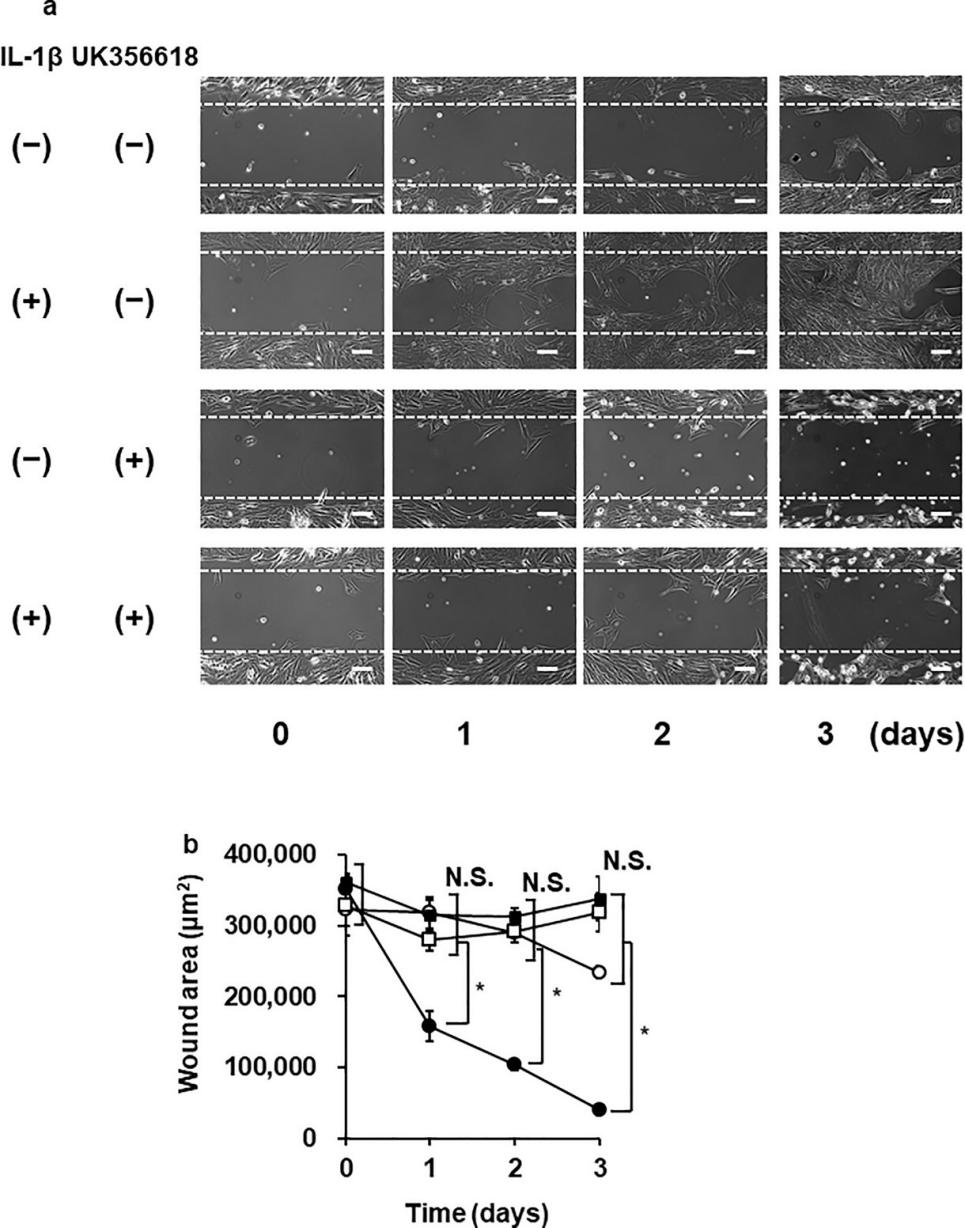
IL-1β-induced cellular migration in dermal fibroblasts. (**a, b**) After the pretreatment with (open and closed square) or without (open and closed circle) the MMP-3 inhibitor UK356618 (2 μM) for 2 h, the cells were incubated with (closed circle and square) or without (open circle and square) 100 pM IL-1β for indicated time. The representative images of the cell migration with 100 pM IL-1β for 0–3 days (**a**) and the time-dependent changes of wound area (**b**) were shown. Values are expressed as the mean ± SE of 3 independent experiments. The F values were 11.23 (**b**, 1 day) 109.93 (**b**, 2 day) and 36.00 (**b,** 3 day). The degrees of freedom were 3 (**b**, 1–3 day). *P < 0.05.

We checked whether MMP-3 implicated in the cellular biological function, such as cellular migration. When the cells were treated with IL-1β (100 pM), cell migration occurred in a time-dependent manner. The MMP-3 inhibitor UK356618 (2 μM, 2 h) significantly inhibited the IL-1β-induced cell migration (Fig 2A and 2B). These observations suggest that IL-1β contributes to cell migration via MMP-3 expression.

### ATF-2 contributes to IL-1β-induced MMP-3 expression

**Fig 3:**
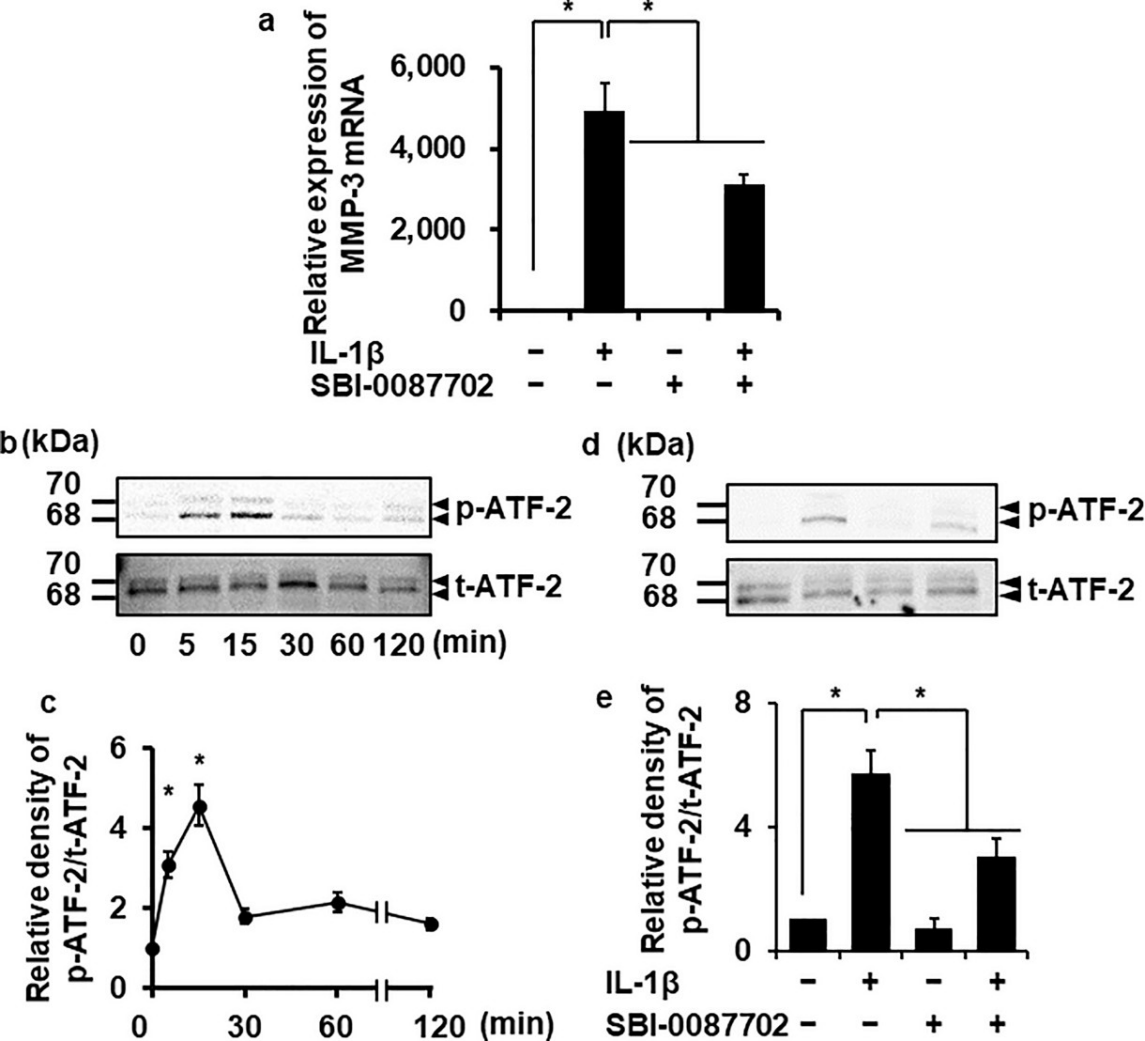
Suppression of IL-1β-induced MMP-3 mRNA expression by an ATF-2 inhibitor and IL-1β-induced ATF-2 phosphorylation. (**a**) After incubation in the presence or absence of the ATF-2 inhibitor SBI-0087702 (10 μM) for 24 h, the cells were stimulated with 100 pM IL-1β or control for 6 h. At the end of the incubation, MMP-3 mRNA expression was measured. (**b, c**) The cells were exposed to 100 pM IL-1β for indicated time intervals. At the end of the incubation, total (t-) and phosphorylated (p-) ATF-2 were detected by western blotting. For the western blotting, cell lysate (10 μg protein) was applied to each lane. Representative results of p- and t-ATF-2 expression (**b**) and the relative density of p-ATF-2 expression in the cells stimulated with 100 pM IL-1β compared to the results at time point 0 (**c**) are shown. (**d, e**) After incubation in the presence or absence of the ATF-2 inhibitor SBI-0087702 (10 μM) for 24 h, the cells were stimulated with 100 pM IL-1β or control for 15 min. At the end of the incubation, t- and p-ATF-2 were detected by western blotting. Representative results of p- and t-ATF-2 expression (**d**) and the relative density of p-ATF-2 expression are shown (**e**). Values are expressed as the mean ± SE of 3 independent experiments. The F values were 53.31 (**a**), 53.50 (**c**) and 17.62 (**e**). The degrees of freedom were 3 (**a**), 5 (**c**) and 3 (**e**). *P < 0.05, compared with 0 h.

The expression of MMPs has been considered to be primarily regulated at the transcriptional level, because most members of the MMP family share common *cis*-elements in their promoter sequences [23–25]. The transcription factor ATF-2 is known to be involved in the regulation of numerous proteins and plays a role in the IL-1β-induced increase in their expression [40, 41]. We sought to establish if this previously observed modulatory effect of ATF-2 occurs in dermal fibroblasts. Fibroblasts pretreated with the ATF-2 inhibitor, SBI-0087702 (10 μM) for 24 h failed to show IL-1β-induced increase in MMP-3 mRNA expression (Fig 3A), suggesting that ATF-2 facilitates this phenomenon.

Then, we examined ATF-2 phosphorylation in cells treated with IL-1β and found it was increased with the peak levels occurring 15 min post-treatment, whereas no change in total-ATF-2 (t-ATF-2) expression was observed. After reaching the peak, the ATF-2 phosphorylation steadily declined to the basal level within 30 min (Fig 3B). These observations indicate that ATF-2 was activated by IL-1β stimulation and is likely to contribute to the effect of IL-1β on MMP-3 expression in dermal fibroblasts. We confirmed that the ATF2 inhibitor SBI-0087702 reduced IL-1β-induced ATF2 phosphorylation (Fig 3D and 3E).

**Fig 4:**
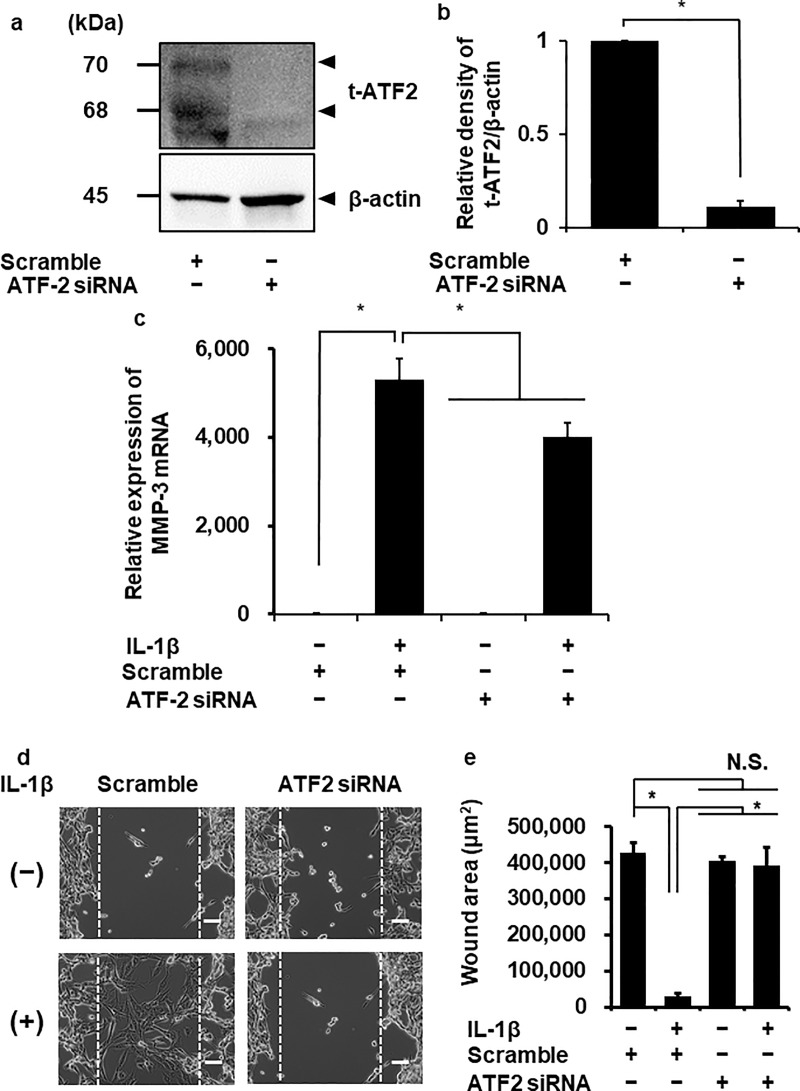
Attenuation of IL-1β-induced MMP-3 mRNA expression in fibroblasts transfected with ATF-2 siRNA. (**a**) In the cells transfected with ATF-2 and scrambled siRNAs, t-ATF-2 and β-actin were detected by western blotting. ATF-2 siRNA-transfection, but not scramble siRNA-transfection, decreased the protein expression of ATF-2. β-actin was used as an internal standard. (**b**) Relative density of ATF-2 protein expression in siRNA-transfected cells, compared to that of scrambled siRNA transfected cells, is illustrated. (**c**) After the transfection with ATF-2 and scrambled siRNAs, the cells were incubated with or without 100 pM IL-1β for 6 h. At the end of the incubation, MMP-3 mRNA expression was determined. TBP was used as an internal standard. IL-1β-induced MMP-3 mRNA expression was attenuated in cells transfected with ATF-2 siRNA compared with those transfected with scrambled siRNA. (**d, e**) After the transfection with ATF-2 or scrambled siRNAs, the cells were incubated with or without 100 pM IL-1β for 3 days. The representative images of the cell migration (**d**) and the calculated wound area (**e**) were shown. Results are presented as mean ± SE from 3 independent experiments. Values are expressed as the mean ± SE of 3 independent experiments. The T values was 31.66 (**b**). The F value was 137.16 (**c**) and 142.01 (**e**). The degrees of freedom were 2 (**b**), 3 (**c**) and 3 (**e**). *P < 0.05.

Next we performed ATF-2 knockdown experiment using siRNA transfection. The protein expression of ATF-2 was significantly reduced in the cells transfected with ATF-2 siRNA, but not in scramble siRNA-transfected cells (Fig 4A and 4B). In the ATF-2 siRNA-transfected cells, IL-1β-induced MMP-3 mRNA expression was attenuated (Fig 4C). We confirmed that IL-1β-induced cellular migration was attenuated in the cells transfected with ATF-2 siRNA (Fig 4D and 4E). These observations suggest that ATF-2 contributes to IL-1β-induced MMP-3 expression.

### ERK signaling pathway is involved in IL-1β-mediated MMP-3 expression

IL-1β has been demonstrated to induce MMP-3 expression via the mitogen-activated protein (MAPK) signaling pathways [42–44], and ATF-2 has also been shown to be regulated via MAPK signaling pathways in response to inflammatory cytokines [40, 41, 45, 46]. Then, as the following study, we examined the relationship between ATF-2 and MAPK signaling pathways.

**Fig 5:**
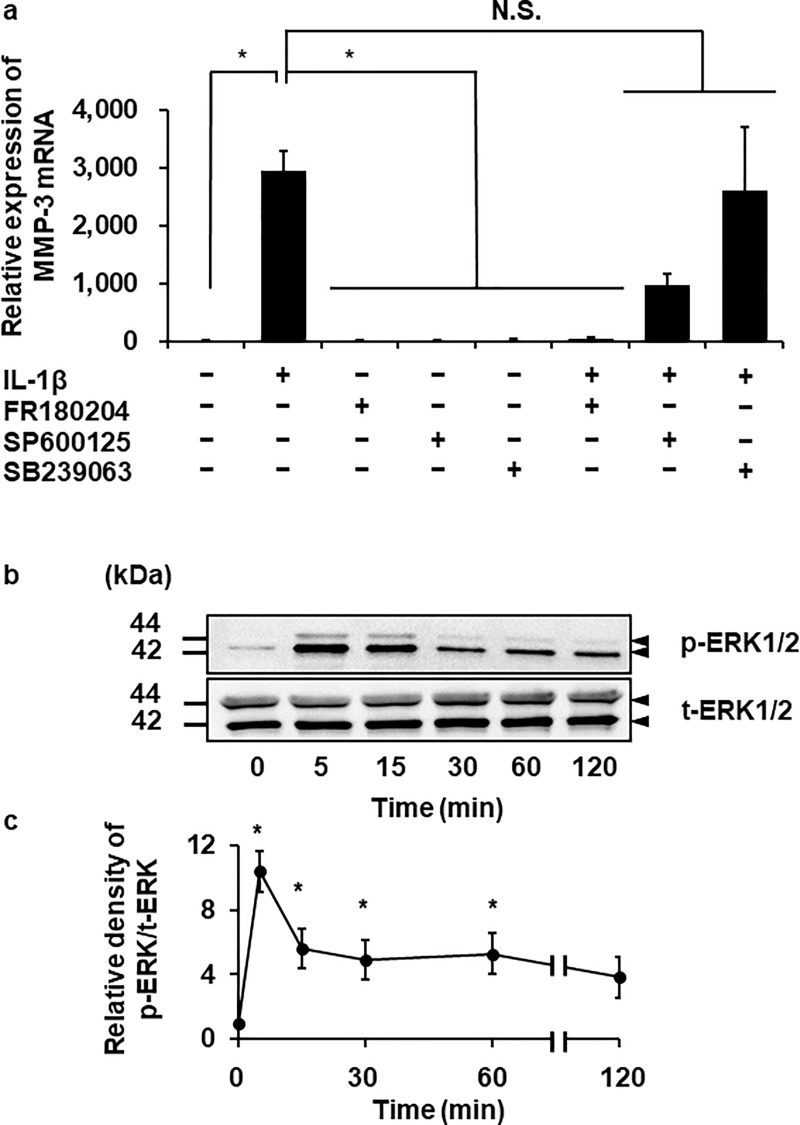
Inhibition of IL-1β-induced MMP-3 mRNA expression by an ERK inhibitor and IL-1β-induced ERK1/2 phosphorylation. (**a**) After incubation in the presence or absence of ERK, p38 and JNK inhibitors (FR180204 (25 μM), SB203963 (20 μM) and SP600125 (10 μM), respectively), for 1 h, the cells were treated with 100 pM IL-1β for 6 h or left untreated. At the end of the incubation, MMP-3 mRNA expression was detected. TBP was used as an internal standard. (**b, c**) The cells were treated with 100 pM IL-1β for indicated time. At the end of the incubation, phosphorylated (p-) and total (t-) ERK1/2 were detected by western blotting. Cell lysate (10 μg protein) was applied to each lane. Representative results of p- and t-ERK1/2 expression (**b**), and the relative density of p-ERK1/2 expression compared to the results at time point 0 (**c**), are shown. Values are expressed as the means ± SE of 3 independent experiments. The F values were 9.68 (**b**) and 16.64 (**c**). The degrees of freedom were 7 (**b**) and 5 (**c**). *P < 0.05, compared with 0 h.

Regarding MAPK signaling on IL-1β-induced MMP-3 expression, we investigated the effects of inhibitors of ERK, c-Jun N-terminal kinase (JNK), and p38 MAPK, which are the enzymes representing the three main MAPK pathway [47, 48]. In the cells treated with the ERK inhibitor FR180204 (25 μM) for 1 h, IL-1β failed to induce MMP-3 mRNA expression, whereas SB203963 (20 μM) and SP600125 (10 μM), inhibitors of p38 MAPK and JNK, respectively, showed less effects on IL-1β-induced MMP-3 mRNA expression (Fig 5A). Since activated MAPKs subsequently phosphorylate and activate downstream targets such as transcription factors and regulators of cell functions, we next examined whether IL-1β activated ERK by detecting the phosphorylation status using immunoblotting with an anti-phospho-ERK1/2 (p-ERK1/2) antibody. The cells stimulated with 100 pM IL-1β showed an increase in p-ERK1/2 but not total-ERK1/2 (t-ERK1/2) expression (Fig 5B and 5C). The maximum phosphorylation levels were observed 5 min after stimulation and then they gradually decreased.

**Fig 6:**
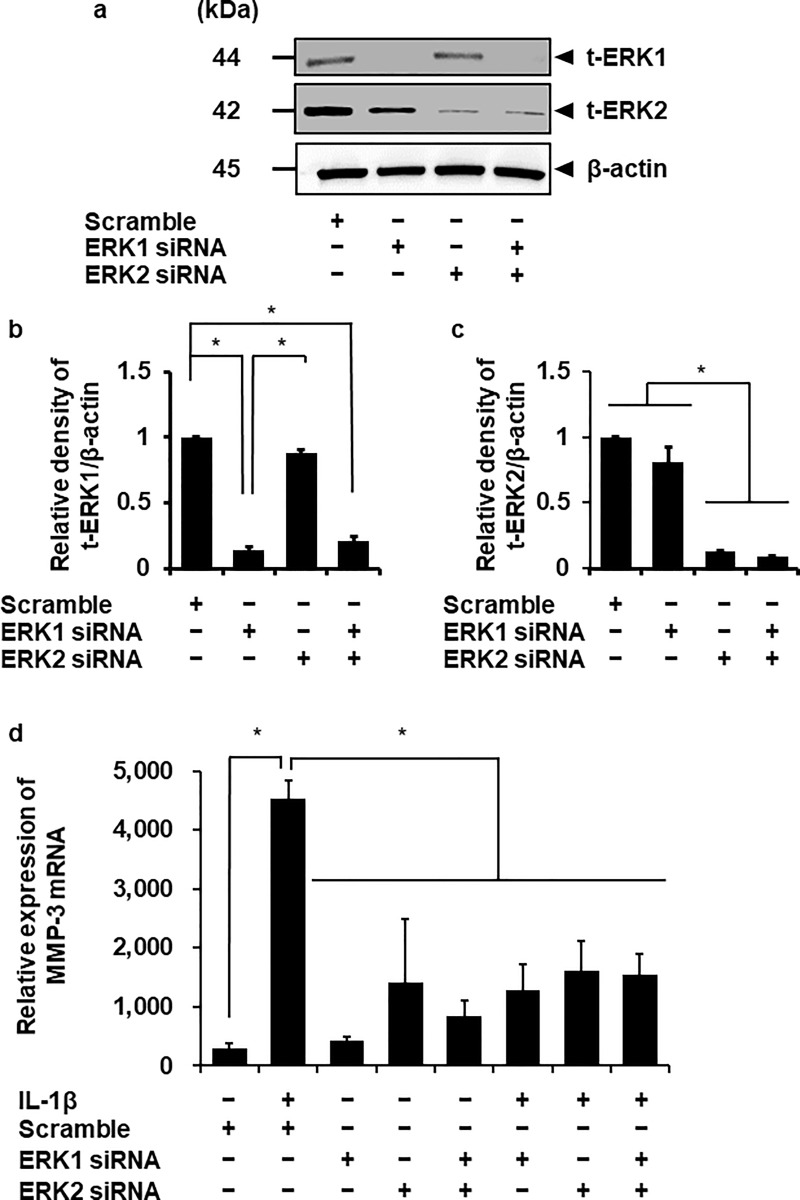
Attenuation of IL-1β-induced MMP-3 mRNA expression in the fibroblasts transfected with ERK1 and ERK2 siRNAs. (**a-c**) In the cells transfected with ERK1, ERK2, and scrambled siRNAs, expression of t-ERK1, t-ERK2, and β-actin was detected by western blotting. ERK1 and ERK2 siRNA-transfection decreased the expression of ERK1 or ERK2, respectively, while scrambled siRNA-transfection did not alter their expression. β-actin was used as an internal standard. Representative results (**a**) and relative density of ERK1 or ERK2 protein expression in siRNA-transfected cells compared to those in scrambled siRNA- transfected cells (**b, c**) are shown. (**d**) After the transfection with ERK1, ERK2 and scrambled siRNAs, the cells were incubated with or without 100 pM IL-1β for 6 h. At the end of the incubation, MMP-3 mRNA expression was determined. TBP was used as an internal standard. ERK1 and 2 siRNA-transfection clearly inhibited IL-1β-induced MMP-3 mRNA expression while scrambled siRNA-transfection did not. The IL-1β-induced MMP-3 mRNA expression was also attenuated in ERK1 and 2 double knockdown cells. Values are expressed as the mean ± SE of 3 independent experiments. The F values were 675.8 (**b**), 10.69 (**c**) and 9.66 (**d**). The degrees of freedom were 2 (**b**), 2 (**c**) and 7 (**d**). *P < 0.05.

**Fig 7:**
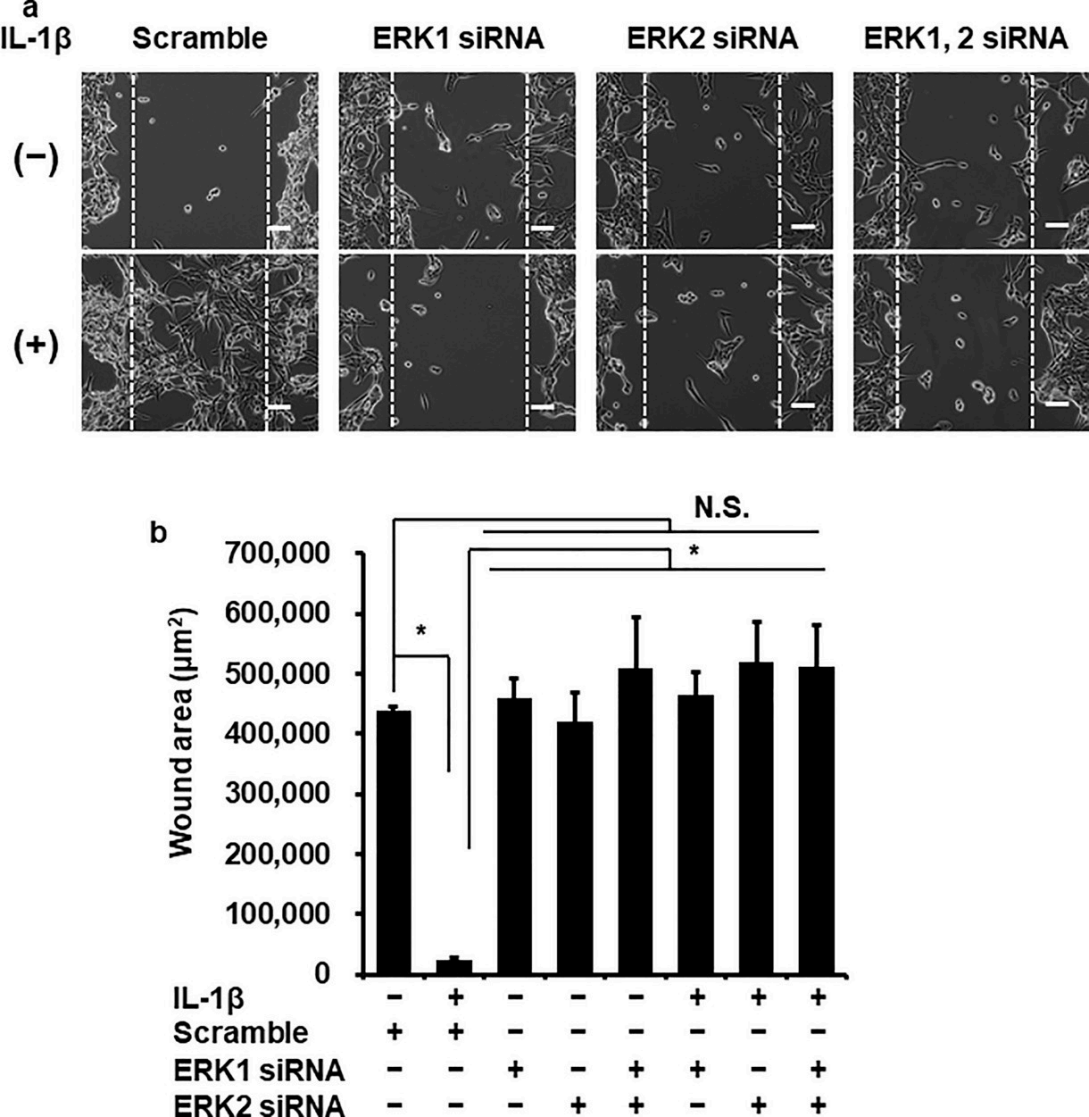
Attenuation of IL-1β-induced cellular migration in the fibroblasts transfected with ERK1 and ERK2 siRNAs. After the transfection with ERK1, ERK2 or scrambled siRNAs, the cells were incubated with or without 100 pM IL-1β for 3 days. The representative images of the cell migration (**a**) and the calculated wound area (**b**) were shown. When the cells were transfected with ERK1 and 2 siRNA, IL-1β-induced cellular migration was clearly attenuated, while scrambled siRNA-transfection did not. The IL-1β-induced celluar migration was also attenuated in ERK1 and 2 double knockdown cells. Values are expressed as the mean ± SE of 3 independent experiments. The F values was 8.40 (**b**). The degrees of freedom was 7 (**b**). *P < 0.05.

To confirm the contribution of ERK1/2 in IL-1β-induced MMP-3 mRNA expression, we performed ERK1/2 knockdown experiment using siRNA transfection. The protein expression of ERK1 or ERK2 was significantly reduced by the transfection with respective siRNAs, but not with scramble siRNA as a control (Fig 6A–6C). IL-1β-induced MMP-3 mRNA expression was attenuated in the ERK1 and ERK2 siRNA-transfected cells compared with the scramble siRNA-transfected cells (Fig 6D). Subsequently, we performed the experiments with ERK1 and ERK2 siRNAs together. In the ERK1 and 2 double-knockdown cells, IL-1β-induced MMP-3 mRNA expression was also attenuated (Fig 6D). We observed that IL-1β-induced cellular migration was attenuated in the cells transfected with siRNA for ERK1 or ERK2. In the ERK1 and 2 double-knockdown cells, IL-1β-induced cellular migration was also attenuated. However, there is no significant difference between the cells transfected with siRNA for ERK1, ERK2 and ERK1/2 (Fig 7A and 7B). These observations strongly suggest that the ERK1/2 activation contributes to the upregulation of MMP-3 mRNA expression induced by IL-1β in dermal fibroblasts.

### ATF-2 contributes to IL-1β-induced MMP-3 expression as a downstream target of ERK1

**Fig 8:**
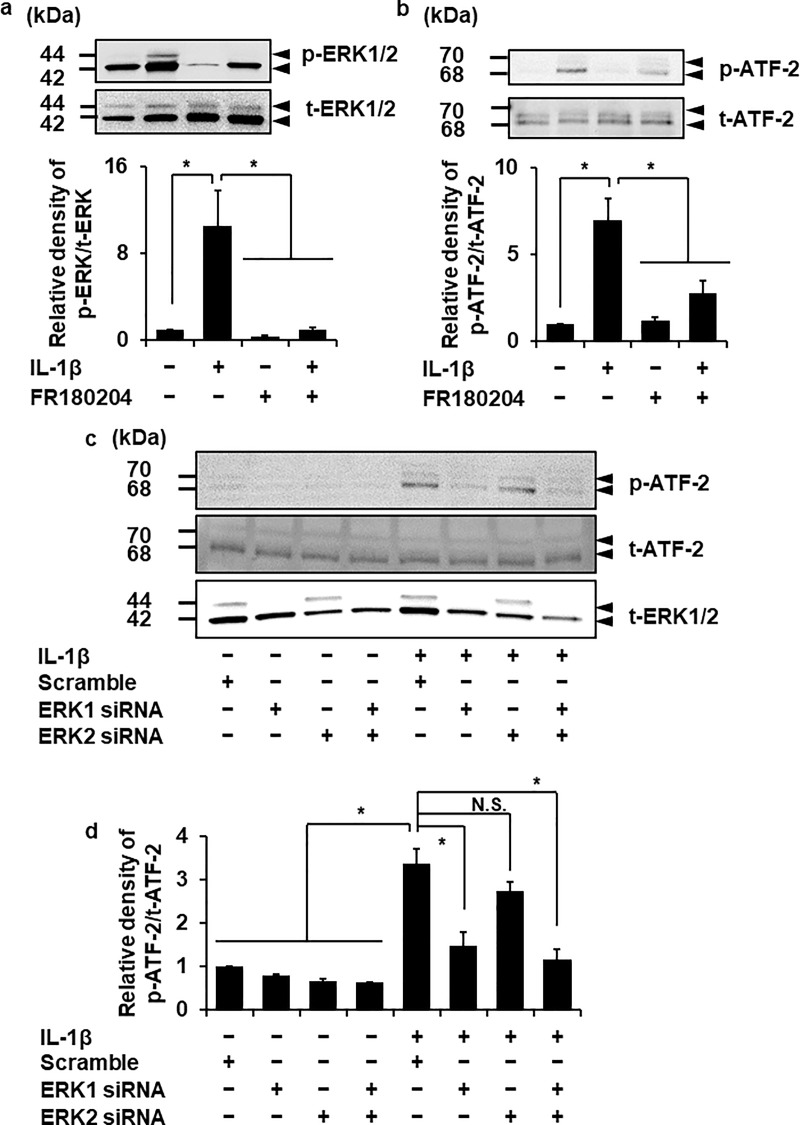
Attenuation of IL-1β-induced ATF-2 phosphorylation in fibroblasts transfected with ERK1 siRNA but not with ERK2 siRNA. (**a, b**) After incubation in the presence or absence of ERK inhibitor FR180204 (25 μM) for 1 h, the cells were treated with 100 pM IL-1β for 15 min. At the end of the incubation, phosphorylated (p-) and total (t-) ATF-2 (**a**) and ERK1/2 (**b**) were detected by western blotting. For the western blotting, cell lysate (10 μg protein) was applied to each lane. Representative results of p- and t-ATF-2 (**a**; upper panel) or p- and t-ERK1/2 expression (**b**; upper panel) and the relative density of p-ATF-2 (**a**; lower panel) and p-ERK1/2 expression (**b**; lower panel) compared to non-treated cells are illustrated. (**c, d**) The cells transfected with ERK1, ERK2 and scrambled siRNAs were incubated in the absence and presence of 100 pM IL-1β for 15 min. Representative results of expression of p- and t-ATF-2 and t-ERK (**c**) and the relative density of p-ATF-2 expression compared to scramble siRNA-transfected cells (**d**) are shown. siRNA-transfection clearly inhibited the IL-1β-induced ATF-2 phosphorylation compared with scramble or ERK2 siRNA-transfection. The IL-1β-induced ATF-2 phosphorylation was also attenuated in ERK1 and 2 double knockdown cells. Values are expressed as the mean ± SE of 3 independent experiments. The F values were 9.16 (**a**), 22.71 (**b**) and 49.48 (**d**). The degrees of freedom were 3 (**a**), 3 (**b**) and 7 (**d**). *P < 0.05.

In the following study, we examined whether ERK signaling regulated the activation of ATF-2 in IL-1β-treated dermal fibroblasts. We first confirmed that the ERK inhibitor FR180204 (25 μM) reduced IL-1β-induced ERK1/2 phosphorylation (Fig 8A). In the cells treated with the ERK inhibitor, the phosphorylation of ATF-2 induced by IL-1β was attenuated (Fig 8B). To examine the contribution of ERK subtypes to ATF-2 phosphorylation, we performed ERK1/2 knockdown experiment. IL-1β-induced ATF-2 phosphorylation was clearly inhibited by ERK1 siRNA transfection, but not by scramble siRNA transfection (Fig 8B and 8D). We also observed that ERK2 siRNA had no effect on IL-1β-induced ATF-2 phosphorylation. Therefore, we examined the effect of ERK1 and ERK2 double knockdown on IL-1β-induced ATF-2 phosphorylation. In the cells transfected with ERK1 and ERK2 siRNAs, IL-1β-induced ATF-2 phosphorylation was reduced compared with a control (Fig 8C and 8D). Taken together, it is most likely that ERK1 signaling is dominantly involved in the activation of ATF-2 in IL-1β-induced MMP-3 expression.

## Discussion

Several observations suggest that MMP-3 has an important role in wound healing [49]. It was shown that the expression of MMP-3 in wound effluent of patients with impaired wound healing was lower than in those with normal wound healing [50]. Additionally, In MMP-3 null mice, delayed wound healing has been demonstrated. Finally, in MMP-3-deficient fibroblasts, the ability of fibroblasts to contract collagen gels was reduced. Moreover, MMP-3 has been also demonstrated to contribute to type I collagenolysis [17]. In this study, we observed that IL-1β failed to induce cellular migration in the presence of UK356618, a specific inhibitor for protease activity of MMP-3, suggesting that the protease activity of MMP-3 plays a crucial role in dermal fibroblasts. Therefore, MMP-3 is considered to have a key role in wound contraction and tissue remodeling during wound repair [51, 52].

In this study, we demonstrated that the ATF-2 inhibitor SBI-0087702 inhibited IL-1β-induced MMP-3 mRNA expression and that IL-1β failed to induce MMP-3 mRNA expression in ATF-2 knockdown cells. Taken together, these observations strongly suggest that ATF-2 is involved in IL-1β-stimulated MMP-3 expression in dermal fibroblasts. In unstimulated cells, ATF-2 is maintained in a transcriptionally inactive form by an intramolecular inhibitory interaction. Namely, the DNA binding domain is folded and binds to the amino-terminal transactivation domain [53]. In response to stimuli, N-terminal phosphorylation has been demonstrated to release the intramolecular inhibition and lead to the activation of ATF-2 [54–56]. Activated ATF-2 regulates gene expression through homo- or hetero-dimerization with other AP-1 family members, such as the CREB, Fos, Maf, or Jun family transcription factors [57, 58]. Additionally, the phosphorylation of ATF-2 has also been demonstrated to control its degradation [59]. It is possible that such mechanisms can influence MMP-3 expression induced by IL-1β, because IL-1β induces ATF-2 phosphorylation in dermal fibroblasts.

MAPK pathways have been reported to phosphorylate ATF-2. In response to stimuli including inflammatory cytokines, JNK and p38 MAPK, phosphorylate ATF-2 at amino acids Thr69 and Thr71 [45, 55, 60]. ERK1/2 was also reported to phosphorylate ATF-2 at Thr71 in the ultraviolet response [61]. In cells stimulated with growth factors such as insulin and epidermal growth factor, the cooperative interaction between ERK and p38 pathways has been suggested as a two-step mechanism, in which the Ras-Raf-MEK-ERK pathway triggers ATF-2 Thr71 phosphorylation, and the Ras-Ral-Src-p38 pathway subsequently stimulates Thr69 phosphorylation [62]. In our study, the ERK inhibitor significantly inhibited IL-1β-induced MMP-3 mRNA expression, but not p38 and JNK inhibitors, and attenuated IL-1β-induced ATF-2 phosphorylation. Therefore, it is likely that ATF-2 Thr71 phosphorylation is involved in IL-1β-induced MMP-3 expression in dermal fibroblasts.

Multiple MAPK signaling pathways are activated by several stimuli including cytokines such as IL-1β [48]. In the previous study, it has been reported that three major MAPK signaling pathways, ERK, JNK, and p38 MAPK, are activated in IL-1β-treated periadipocytes [63]. In cardiac fibroblasts, IL-1β-induced p38 MAPK activation was reported to contribute to MMP3 expression [64]. In human dermal fibroblasts, ultraviolet B induces the activation of JNK and p38 MAPK [65]. We previously reported that IL-1β activated ERK, JNK and p38 MAPK and subsequently induced COX-2 expression in feline synovial fibroblasts [66]. On the other hand, in canine dermal fibroblasts, we observed that IL-1β induced COX-2 expression via the activation of ERK signaling [37]. These observations strongly suggest that the response of MAPKs is highly dependent on the cellular context. ERK1 and ERK2, ERK isoforms, possess 83% amino acid identity and are co-expressed in most tissues [67, 68]. These two isoforms are generally co-activated in cells stimulated with multiple extracellular stimuli [69–71]. Recently, the functional differences between the two isoforms were demonstrated using antisense techniques or siRNA transfection [66, 68, 72–76]. Here, we performed ERK-knockdown experiments by treatment with ERK isoform-specific siRNA. Both in ERK1 and ERK2 knockdown cells, IL-1β-induced MMP-3 mRNA expression was attenuated. However, IL-1β-induced ATF-2 phosphorylation was attenuated in ERK1 knockdown cells, but not in ERK2- knockdown cells. These results suggest that ERK1 contributes to MMP-3 expression via transcriptional activation of ATF-2 in dermal fibroblasts. On the other hand, transcriptional regulation of MMP-3 via ERK2 pathway remained unclear, which appears to cause the partially inhibitory effect of ATF-2 inhibitor on IL-1β-induced MMP-3 mRNA expression.

In cells treated with an ATF-2 inhibitor and transfected with ATF-2 siRNA, IL-1β-induced MMP-3 mRNA expression was partially inhibited. These results suggest that other transcription factors may probably contribute to IL-1β-induced MMP-3 mRNA expression as well as ATF-2. Genes of MMP family including MMP-3 contain several *cis*-elements in their promoter sequences, which allow a tight control of cell-specific MMP gene expression by a diverse set of *trans*-activators, such as polyomavirus enhancer-A binding protein-3 (PEA3) and NF-κB, as well as ATF-2 [24, 77]. In this study, in cells treated with an ERK1/2 inhibitor and transfected with ERK1 or 2 siRNA, the effect of IL-1β on MMP-3 mRNA expression was inhibited. However, the IL-1β-induced ATF2 phosphorylation was attenuated in the cells transfected ERK1 siRNA, but not ERK2 siRNA. Therefore, ERK2 pathway appears to be necessary for activating other transcription factors in fully MMP-3 expression induced by IL-1β. Currently our laboratory is trying to further clarify the role of such transcription factors regulated by ERK2.

## Conclusions

**Fig 9:**
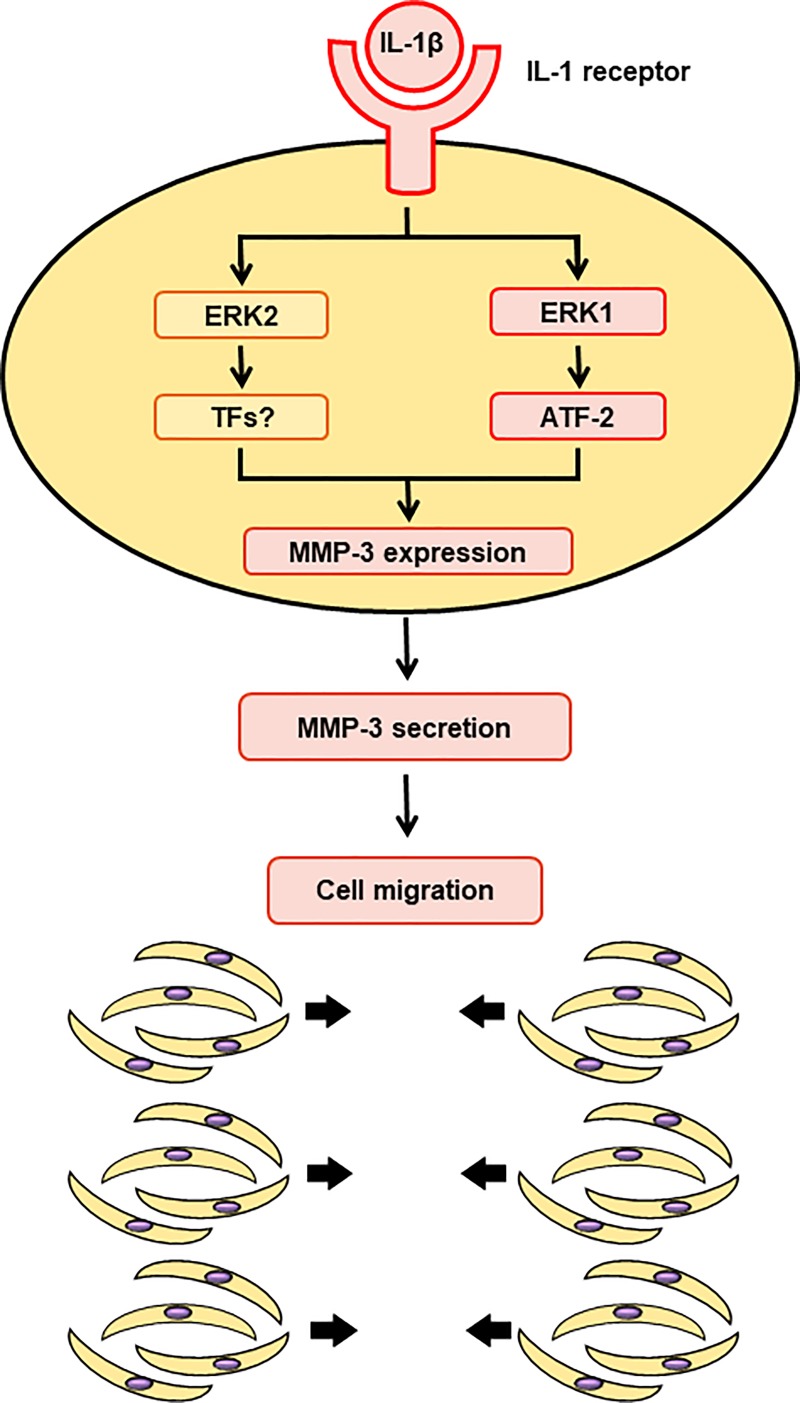
ERK1/ATF-2 signaling axis contributes to IL-1β-induced MMP-3 expression in dermal fibroblasts. In dermal fibroblasts, ERK1 contributes to IL-1β evoked the activation of ERK1/ATF-2 signaling axis, which contributes to cellular migration via MMP-3 expression. ERK2 pathway appears to be necessary for activating other transcription factors in fully MMP-3 expression induced by IL-1β.

In conclusion, the findings of this study are significantly helping the understanding of the ERK1 MAPK signaling axis, and the role of transcription factor ATF-2, in respect to IL-1β-induced MMP-3 expression in dermal fibroblasts (Fig 9). Such mechanism regulating MMP-3 expression, appears to be important for the occurrence of wound healing in the skin.
